# Home-based monitoring of persons with advanced Parkinson’s disease using smartwatch-smartphone technology

**DOI:** 10.1038/s41598-023-48209-y

**Published:** 2024-01-02

**Authors:** Tsviya Fay-Karmon, Noam Galor, Benedetta Heimler, Asaf Zilka, Ronny P. Bartsch, Meir Plotnik, Sharon Hassin-Baer

**Affiliations:** 1 Movement Disorders Institute, Department of Neurology, Sheba Medical Center, Ramat Gan, Israel; 2 Center of Advanced Technologies in Rehabilitation, Sheba Medical Center, Ramat Gan, Israel; 3 Department of Physics, Bar-Ilan University, Ramat Gan, Israel; 4 Department of Physiology and Pharmacology, Faculty of Medicine, Tel Aviv University, Tel Aviv, Israel; 5 Sagol School of Neuroscience, Tel Aviv University, Tel Aviv, Israel; 6 Department of Neurology and Neurosurgery, Faculty of Medicine, Tel Aviv University, Tel Aviv, Israel

**Keywords:** Parkinson's disease, Signal processing

## Abstract

Movement deterioration is the hallmark of Parkinson’s disease (PD), characterized by levodopa-induced motor-fluctuations (i.e., symptoms’ variability related to the medication cycle) in advanced stages. However, motor symptoms are typically too sporadically and/or subjectively assessed, ultimately preventing the effective monitoring of their progression, and thus leading to suboptimal treatment/therapeutic choices. Smartwatches (SW) enable a quantitative-oriented approach to motor-symptoms evaluation, namely home-based monitoring (HBM) using an embedded inertial measurement unit. Studies validated such approach against in-clinic evaluations. In this work, we aimed at delineating personalized motor-fluctuations’ profiles, thus capturing individual differences. 21 advanced PD patients with motor fluctuations were monitored for 2 weeks using a SW and a smartphone-dedicated app (Intel Pharma Analytics Platform). The SW continuously collected passive data (tremor, dyskinesia, level of activity using dedicated algorithms) and active data, i.e., time-up-and-go, finger tapping, hand tremor and hand rotation carried out daily, once in OFF and once in ON levodopa periods. We observed overall high compliance with the protocol. Furthermore, we observed striking differences among the individual patterns of symptoms’ levodopa-related variations across the HBM, allowing to divide our participants among four data-driven, motor-fluctuations’ profiles. This highlights the potential of HBM using SW technology for revolutionizing clinical practices.

## Introduction

Parkinson’s disease (PD) is the second most prevalent neurodegenerative disorder worldwide^1^. When an individual is diagnosed with PD, a substantial proportion of dopamine (DA) producing neurons in the nigrostriatal pathways have been lost, causing hypokinetic motor symptoms such as bradykinesia, rigidity, tremor and others. Most of these motor symptoms can be substantially improved with dopaminergic medications, especially levodopa (l-dopa), the precursor of DA. Although initially effective for management of motor symptoms, chronic administration of l-dopa is eventually complicated by motor fluctuations (MF), including predictable or unpredictable ‘OFF’ times (periods of recurrence of PD symptoms when the medication effects wear off). Another motor phenomenon observed with long-term l-dopa treatment is the emergence of abnormal involuntary movements (typically choreiform or dystonic) commonly affecting the facial muscles, neck, upper and lower limbs and trunk, termed levodopa-induced dyskinesia (LID).

Accurate assessment of motor dysfunction in PD is challenging, especially when MF and LID are being evaluated. In clinical trials and clinical practice, the most common measurement instrument presently used to assess PD-associated motor impairment and dysfunction is the revised version of the Unified PD rating scale [the International Parkinson and Movement Disorder Society )MDS(-UPDRS]^2^. Specifically, sections III and IV address the severity of PD motor signs as well as the duration and severity of both off periods and LID. The drawbacks of this scale include its semi-quantitative rating; it is time-consuming and rater-dependent^3^. Furthermore, it only assesses the patient’s condition in the last week and motor symptoms assessed during the clinic visit, and thus cannot provide any reliable information on the long period in-between medical visits. Another standard instrument used to evaluate MF and LID is PD home diaries, in which patients report their motor state over a 24-h period using pen and paper. The most established patient diary is the Hauser Diary^4^ where patients report every half hour during the day, if they were asleep, in ‘OFF’, in ‘ON’ without dyskinesia, ‘ON with non-troublesome dyskinesia’ or ‘ON with troublesome dyskinesia’. Filling the diary requires rigorous patient training; therefore, this tool's effectiveness depends on patients' collaboration and meticulousness. Recall bias and fatigue may easily hamper the accuracy of the diaries. Furthermore, with this approach, LID often fail detection, as many patients are unaware of their presence^5^. Finally, impaired cognition, impulsivity, apathy and depression may all contribute to inaccurate self-reporting, as they can lead to patients’ underestimation or overestimation of their motor symptoms and MF^6^.

All the aforementioned issues are very problematic for clinical research as well as for clinical practice. The accuracy of patient report is critical, as clinicians rely on it to assess disease impact on his/her daily life, and monitor presence and severity of motor and non-motor symptoms along the day, adherence and response to medical treatment and more. Furthermore, the clinicians need the information in order to provide a proper recommendation for a modification of patients’ dopaminergic treatment for improving outcomes.

Due to shortage of medical resources, PD patients usually visit their neurologist at the clinic, no more than 2–3 times a year. The visits are time-limited and may leave some issues unattended, regarding various PD symptoms and overall health^7–9^. Moreover, the lack of continuity and objectivity of measures of PD symptoms and functionality during the period between clinic visits may lead to imprecision and potential biases in approaching clinical problems and goal definitions, particularly regarding PD-related motor states. This may adversely affect the decision-making process and the prescribed treatment plan.

Digital health technology, including wearable and environmental sensors, video cameras and other electronic tools, has provided new opportunities for measuring movement abnormalities of patients with PD^8,9^. Sensors and ad-hoc algorithms for signal interpretation offer a real-time method to objectively and accurately measure motor symptoms and to provide continuous remote health monitoring of PD patients. Such collected data may capture a complete picture of PD motor symptoms and patients’ functionality over long periods, during real-life experiences, and following interventions. It can provide reliable information, which can be helpful in patients’ care and therapeutic or observational clinical studies.

Previous studies have shown that up to 50% of PD patients have uncontrolled motor symptoms^10,11^, and there is hope that new monitoring technology may lead the way to more favorable outcomes. For this reason, the MDS Task Force on Technology suggested a roadmap for the implementation of wearable technology in the everyday life of PD patients, ultimately becoming common clinical practices aiming at improving the outcomes of PD treatments^11^.

Within this framework, the feasibility of using smartphones and their embedded accelerometer sensors for the assessment of PD motor symptoms has been demonstrated in previous studies^12,13^. Zhan et al. developed a machine-learning approach that measured a smartwatch (SW)-derived objective ‘PD severity score’, using accelerometer data, from five different activities (e.g., the inter-tap interval from the finger-tapping activity)^14^. Further studies proved that combining wearable sensors with electronic diaries is feasible for monitoring PD patients in daily life^15^. Furthermore, previous studies implemented home-based monitoring (HBM) of various motor symptoms (mainly tremor, bradykinesia and dyskinesia) in PD patients relying on these SW technologies^16–19^. Across studies, the HBM period lasted for a variable amount of time ranging from 1 week to several months. One of these studies assessed the effectiveness of wearable sensors for determining the on/off state of PD patients suffering from MF^20^. One platform called the PKG^®^ System consisting of a wrist-worn SW logger using proprietary algorithms, has been validated for the measurement of bradykinesia, rest tremor and dyskinesia as well as response to l-dopa^21,22^. The algorithm used in the PKG System recognized bradykinesia as movements made with lower acceleration and amplitude, and with longer intervals between movement, and dyskinesia as movements of normal amplitude and acceleration, but with shorter periods without movement. The algorithm predicted the UPDRS III score with a margin of error similar to the inter-rater limits. Moreover, the PKG System was shown to provide helpful information to the clinician, which was translated into statistically significant improvements in the MDS-UPDRS total, parts III and IV scores, compared to those with conventional assessment^23^.

Another group developed the “Motor fluctuations Monitor for Parkinson’s Disease” (MM4PD) system, that used SW inertial sensors to continuously track fluctuations in resting tremor and dyskinesia. The MM4PD measurements correlated to clinical evaluations of tremor severity (ρ = 0.80), and matched to expert ratings of the presence of dyskinesia (P < 0.001) during in-clinic tasks. MM4PD captured symptom changes in response to treatment, that matched the clinician's expectations in 94% of evaluated subjects^16^. Another study successfully captured daily MFs and short- and long-term responses to therapy changes, using SW-based assessments and sensors^19^.

In this work we implemented a 2-week HBM using the Intel^®^ Pharma Analytics Platform (i.e., SW with a dedicated app, SWA; see “Methods” and Fig. 6 for the summary of the experimental protocol), allowing the analysis of accelerometer/gyroscope extracted data from the wrist of participants. Specifically, from this sensor we continuously collected passive data (tremor, dyskinesia, level of activity using dedicated algorithms; see “Methods”) and active data, i.e., three assessments (time-up-and-go, finger tapping, hand tremor and hand rotation) carried out every day twice: once in OFF and once in ON levodopa periods (see “Methods”). This platform has been already successfully used for monitoring PD patients^24,25^. We enrolled a relatively homogeneous subgroup of advanced PD patients suffering from significant MFs and LID for the study. We aimed to characterize the properties of motor symptoms more thoroughly (i.e., rest and postural tremor; dyskinesia) and related fluctuations using the platform mentioned above and comparing accelerometer/gyroscope extracted data containing motor symptoms’ signatures with in-clinic assessments, home diaries, and a series of other patient-reported outcomes (PROs; see “Methods” for further details). Our final aim was to directly address the possibility of utilizing the HBM approach to obtain detailed and personalized motor symptoms’ profiles, and related individualized MF signatures, across the day(s) and within real-life conditions, taking into consideration both l-dopa intakes and individual activity levels.

## Results

Twenty-four patients were screened and recruited to the study. Three patients were excluded, and their data was not included in the analysis (two patients withdrew consent and did not start the HBM phase, and one patient did not have discernable ON–OFF episodes and did not complete the 2-week HBM period); Data obtained from 21 patients (17 males, mean age: 66.1 ± 6.9) was included in the analysis. See Table 1 for details on demographics and clinical characteristics of participants.

**Table 1:** Advanced Parkinson’s disease participant's characteristics.

Group characteristics		Range
Participants (males), N	21 (17)	
Age, years (mean ± sd)	66.1 ± 6.9	52–78
Age at PD onset, years (mean ± sd)	55.8 ± 8.9	36–71
Disease duration, years (mean ± sd)	10.3 ± 4.6	5–22
Patients according to handedness (right/left), N	17/4	
Patients motor symptom predominance (right/left/symmetrical), N	5/15/1	
Hand with SW (right/left), N	6/15	
MMSE score (mean ± sd)	28.6 ± 1.5	26–30
MoCA score (mean ± sd)	24.2 ± 3.2	19–30
Number of daily l-dopa doses (mean ± sd) (median)	5.1 ± 1.4 (5)	3–9
l-dopa daily dose, mg (mean ± sd)	813 ± 345	400–1590
l-dopa equivalent daily dose, mg (mean ± sd)	1013 ± 387	500–1990
Patients at Hoehn and Yahr stage—OFF 1/2/3, N	0/13/8	1–3
Patients at Hoehn and Yahr stage—ON 1/2/3, N	4/16/1	1–3
MDS-UPDRS part 1 (mean ± sd)	9.3 ± 6.3	1–22
MDS-UPDRS part 2—OFF (mean ± sd)	17.6 ± 8.1	5–29
MDS-UPDRS part 2—ON (mean ± sd)	8.5 ± 6.2	0–20
MDS-UPDRS part 3—OFF (mean ± sd)	44.5 ± 15.3	20–74
MDS-UPDRS part 3—ON (mean ± sd)	24.8 ± 12.4	1–57
MDS-UPDRS part 4 (mean ± sd)	7.8 ± 2.6	2.6–12
PDSS2 score (mean ± sd)	21.7 ± 11.8	3–47
PDQ-39 Score (mean ± sd)	54.9 ± 25.8	7–123
ESS (mean ± sd)	7.5 ± 5	1–17

Averages of all collected information during the clinic visits are summarized in this table.

*MMSE* Mini-mental status examination, *MoCA* The Montreal Cognitive Assessment, *MoCA* MDS-UPDRS Movement Disorder Society‐sponsored revision of the Unified Parkinson's Disease Rating Scale (MDS‐UPDRS), *PDSS2 score* Parkinson's disease sleep scale, *ESS* Epworth Sleepiness Scale, *PDQ-39* Parkinson’s Disease Questionnaire.

### Compliance with the study protocol and medication intake

Overall the compliance using the system during the 2-week HBM period was good, with most subjects (81%) wearing the SW for more than 12 h daily, throughout the tested period (see Fig. S1). Most patients (71%) filled their motor task reports with a small delay of up to 30 min. Furthermore, the majority (86%) had acceptable compliance regarding the execution of the daily motor tasks, with no more than 2 missed motor tasks during the HBM period. Most (76%) had good compliance with the daily questionnaire, with no more than 1 missed query. 81% also showed good compliance with the medication intake reports. According to the medication intake diaries provided by the SWA, most patients (86%) had very good compliance to medication intake and skipped only a few doses; however, most patients had a delay in intake time of some of the doses when compared to the recommended time set by the physician (see Fig. S1).

Compliance results are summarized in Table S1.

### Algorithm validation (SWA vs clinicians)

Validation results are summarized in the table below (see Table 2). For additional details, see Supplementary Materials.

**Table 2:** In-clinic motor tasks results: correlations between clinician and SWA scores.

	Correlation in OFF	Correlation in ON
Rest tremor (clinician's score vs SWA score)	rho = 0.74 (p < 0.001)	rho = 0.58 (p = 0.01)
Postural tremor (clinician's score vs SWA score)	rho = 0.64 (p < 0.001)	rho = 0.57 (p = 0.01)

In-clinic motor tasks results—correlations between the SWA scores and the clinician’s scores for tremor.

### MDS-UPDRS and sensor-data-correlations

The main results regarding the correlation between the MDS-UPDRS parts 2, 3, and 4 with the SWA scores for tremor, dyskinesia, and activity are described in Table 3. For more details, see Supplementary Materials.

**Table 3:** Correlation between MDS-UPDRS items and SWA tremor, dyskinesia and motor fluctuation measures.

MDS-UPDRS items	SWA score	Correlation
Score of item # 2.10 tremor over the past week	Overall tremor	rho = 0.64 (p = 0.003)
Combined score of items # 3.15, 3.16, 3.17: postural, kinetic and rest tremors, respectively	Overall tremor	rho = 0.62 (p = 0.004)
Score of item # 3.15 (postural tremor)	Overall task tremor—posture	rho = 0.49 (p = 0.02)
Score of item # 3.17 (rest tremor) score	Overall task tremor—rest	rho = 0.43 (p = 0.05)
Score of item # 4.3 (Time spent in OFF state)	Overall activity	rho = -0.47 (p = 0.04)
Score of item # 4.1 (time spent with dyskinesia)	Overall dyskinesia	rho = 0.4 (p = 0.09)
Score of item # 4.2 (Functional impact of dyskinesia)	Overall dyskinesia	rho = 0.47 (p = 0.04)
Score of item # 4.3 (Time spent in OFF state)	Overall dyskinesia	rho = − 0.35 (p = 0.14)

As can be seen in Table 3, positive correlations between the SWA obtained HBM-period daily measures, and the MDS UPDRS scores were found for tremor (high correlation), activity level and dyskinesia (moderate correlation).

### Daily symptom diary

**Figure 1:**
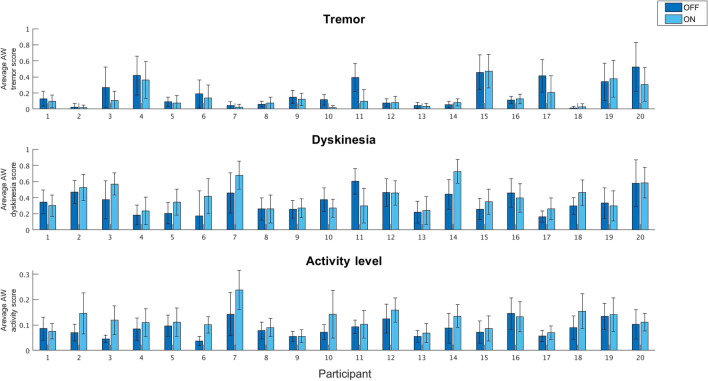
Extracting the symptoms measured by the SWA based on the subjective reports regarding their ON vs OFF states in the daily symptoms’ diaries. In each graph, we depict the individual average SWA measures for tremor, dyskinesia and activity level according to the OFF (dark blue bars) vs ON states (light blue bars) reported by the participants. Top panel: average tremor score. Middle panel: average dyskinesia score. Bottom panel: average activity score. Error bars depict the standard errors.

A summary of the analyses aimed at documenting potential differences in the motor symptoms scores recorded by the SWA based on the participants’ self-reported l-dopa state (i.e., ON vs OFF) is reported in Table 4 and Fig. 1. For detailed analyses, see Supplementary Materials.

**Table 4:** ON vs OFF comparisons of symptoms’ presence based on daily symptoms diary and corresponding SWA recorded signals.

	*t*-test comparing overall symptoms’ scores in ON vs OFF states	Participants [%] with higher symptoms in either ON or OFF states	Participants [%] that had a significant difference between ON and OFF
Tremor	p = 0.02	65% > OFF	38%
Dyskinesia	p = 0.1	65% > ON	61%
Activity	p < 0.01	90% > ON	45%

Comparing SWA recorded signals based on the subjective reports in the daily symptoms diary. The difference in tremor, dyskinesia and activity level recorded by the SWA between ON and OFF states as reported in the daily symptom diary.

### Daily home motor tasks in OFF and ON

Results for the tremor motor tasks are summarized in Table 5, and the results for the finger tapping and TUG3m test are in Table 6. For detailed analysis, see Supplementary Materials.

**Table 5:** Daily motor tasks results—tremor.

	Percent of participants showing tremor [%]	Participants [%] that had significantly more tremor in OFF
Rest tremor	66.67%	21%
Postural tremor	28.5%	16%

Daily motor tasks—tremor results. The percent of participants that showed tremor in the rest and postural tremor tests separately, and the percent of participants that showed more tremor in OFF vs ON in the rest and postural tests separately.

**Table 6:** Daily motor tasks results—finger tapping test and TUG3m.

	Overall OFF score	Overall ON score	OFF vs ON	Participants that performed better in ON vs OFF
Finger tapping test—valid taps	24.6 ± 8.2	34.4 ± 6.6	p < 0.001	90.5% (N = 19)
Finger tapping test—time between taps [s]	0.4 ± 0.13	0.29 ± 0.06	p < 0.001	86% (N = 18)
TUG3m—time [s]	21.68 ± 4.42	17 ± 3.6	p < 0.001	95% (N = 20)

Results of the daily motor tasks: finger tapping test and the TUG3m test. The overall score of the motor tasks in OFF and ON, and the percent of participants that performed better in ON tasks vs OFF tasks.

Note that Table S2 shows detailed results regarding variation over time in the performance of the motor tasks across participants, during the whole HBM period. Results show the lowest variability in the TUG test and in the postural tremor test.

### Individualized motor fluctuations’ patterns: raster plots

**Figure 2:**
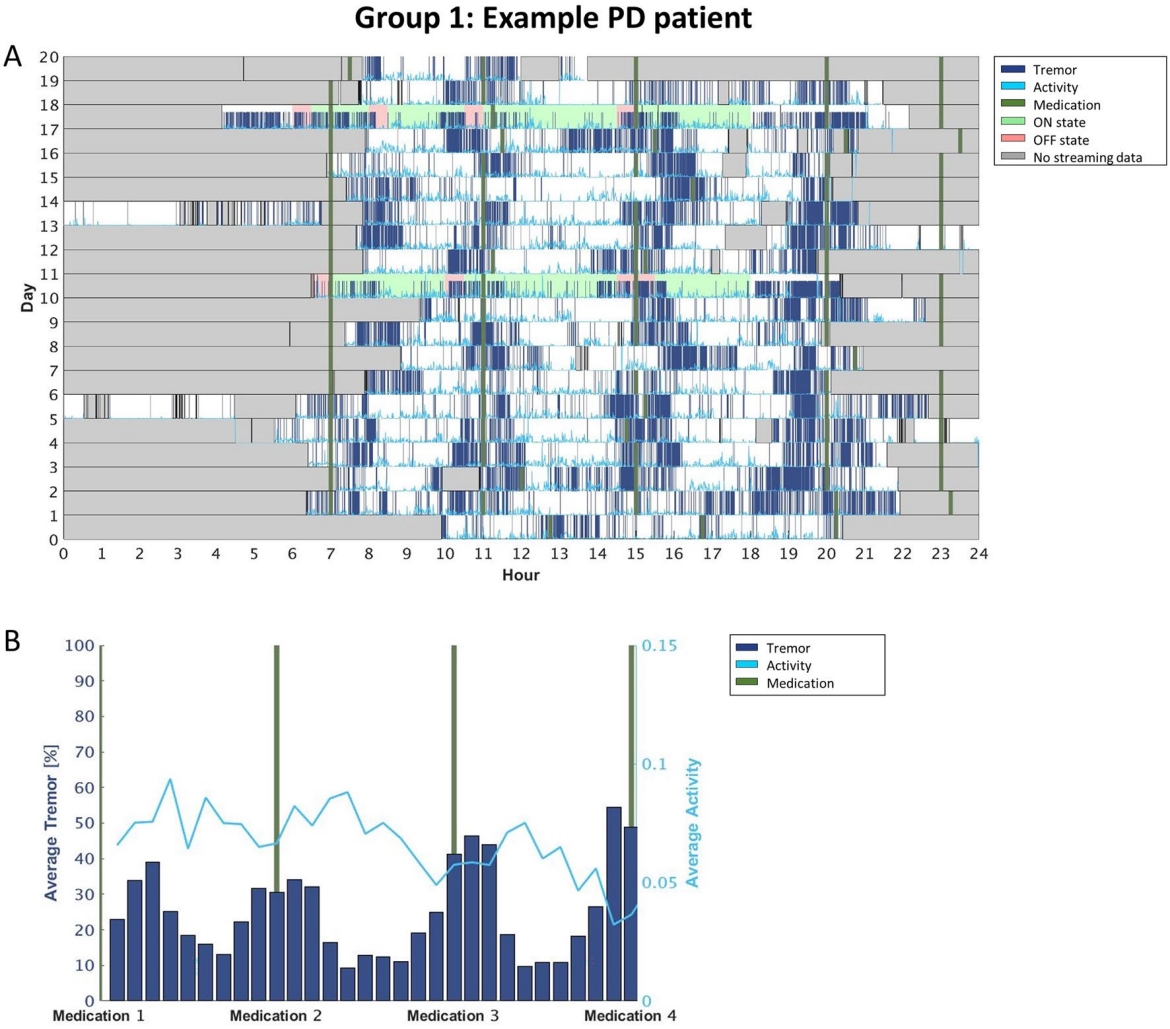
Group 1: A participant whose tremor fluctuates with the times (s)he took his/her medications. (**A**) The raster plot, (**B**) the averaged tremor and activity level scores when scaling the medications intake times. As depicted in the figure, the participant had more tremor around the time of his/her scheduled medication intake (i.e., when l-dopa effects were wearing off) compared to the time between intakes (i.e., during the ON l-dopa state). Additionally, the activity level was the highest in the time between medications, though it tended to decrease throughout the day, reaching its lowest rate at the end of the day.

**Figure 3:**
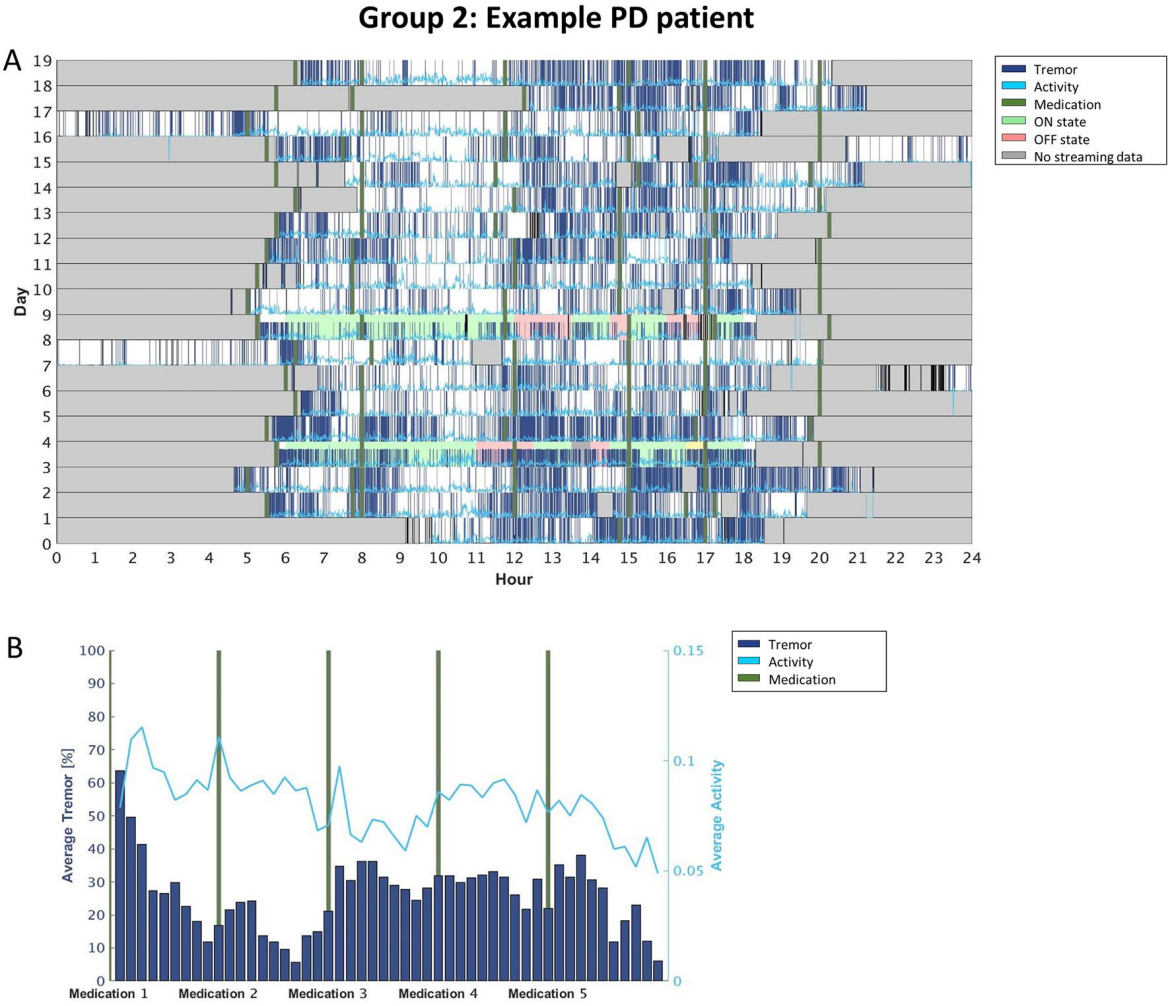
Group 2: A participant showing significantly less tremor in the morning compared to the rest of the day. (**A**) The raster plot, (**B**) the averaged tremor and activity level scores when scaling the medications intake times. As depicted in the figure, the tremor tended to fluctuate around the medications’ intake times in the mornings, but independently of the medications, this participant suffered from persistent tremor after mid-day. However, note that during the 2-days of the symptom diaries (s)he reported OFF periods only in the afternoon, therefore potentially confirming stronger symptoms during this part of the day. Additionally, the activity level of this participant did not change neither according to medications' intake times nor to the tremor absence or presence.

**Figure 4:**
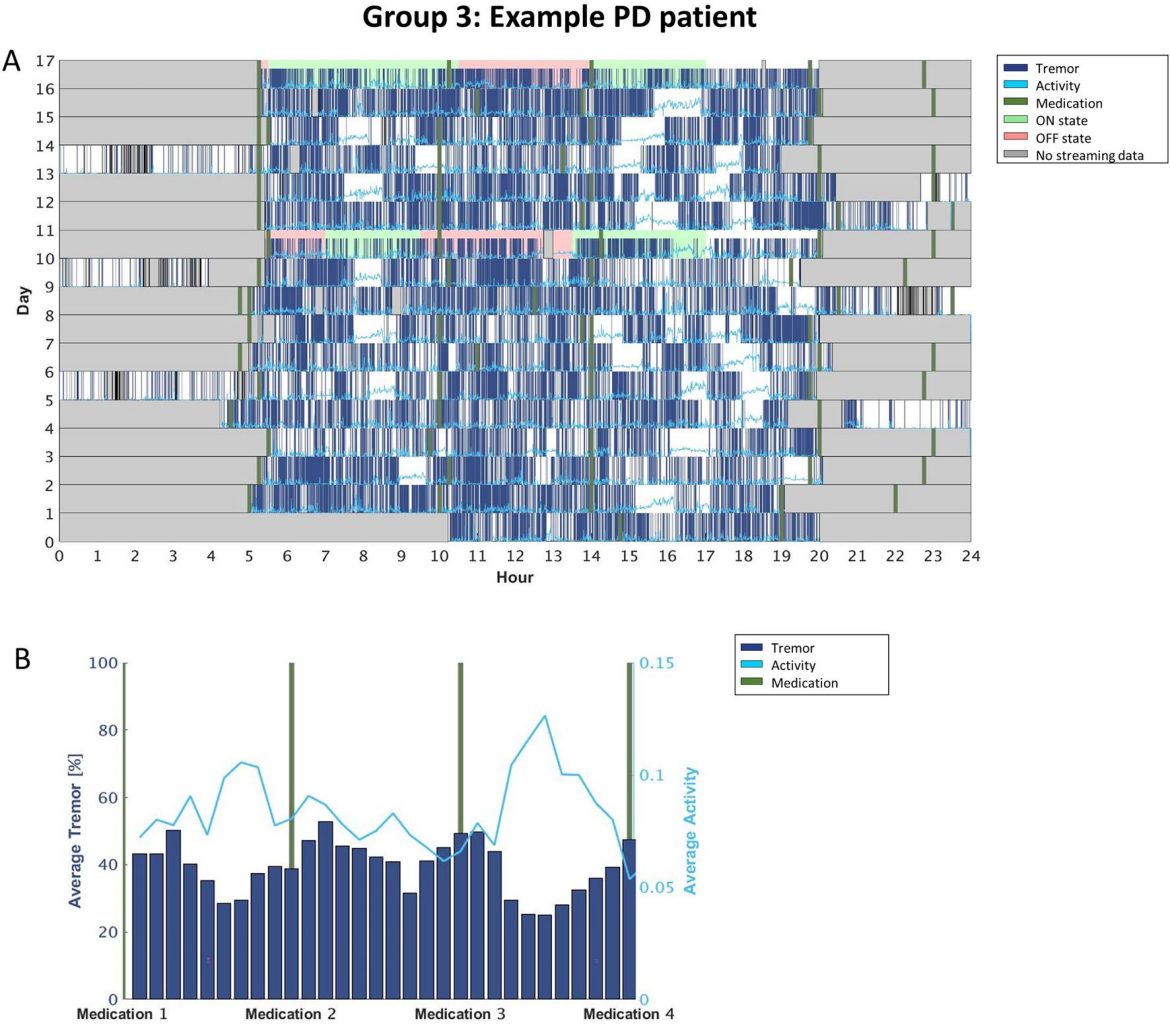
Group 3: A participant with abundant tremor throughout the day (almost all the time). (**A**) The raster plot, (**B**) the averaged tremor and activity level scores when scaling the medications intake times. The tremor did not fluctuate around the times of medication intake, but there were periods between medications where the participant had no tremor. During these times, the activity level was at its highest rate. Note that also, when looking at the patient’s reports during the 2-day symptom diaries, we did not observe any relation between ON/OFF states and presence/absence of tremor. As depicted in (**A**) the participant did not wear the SW after the fourth medication intake during most of the HBM period. Hence, in (**B**) we averaged the tremor-detected percent and the activity level only including the bins between the first and forth medication intakes.

**Figure 5:**
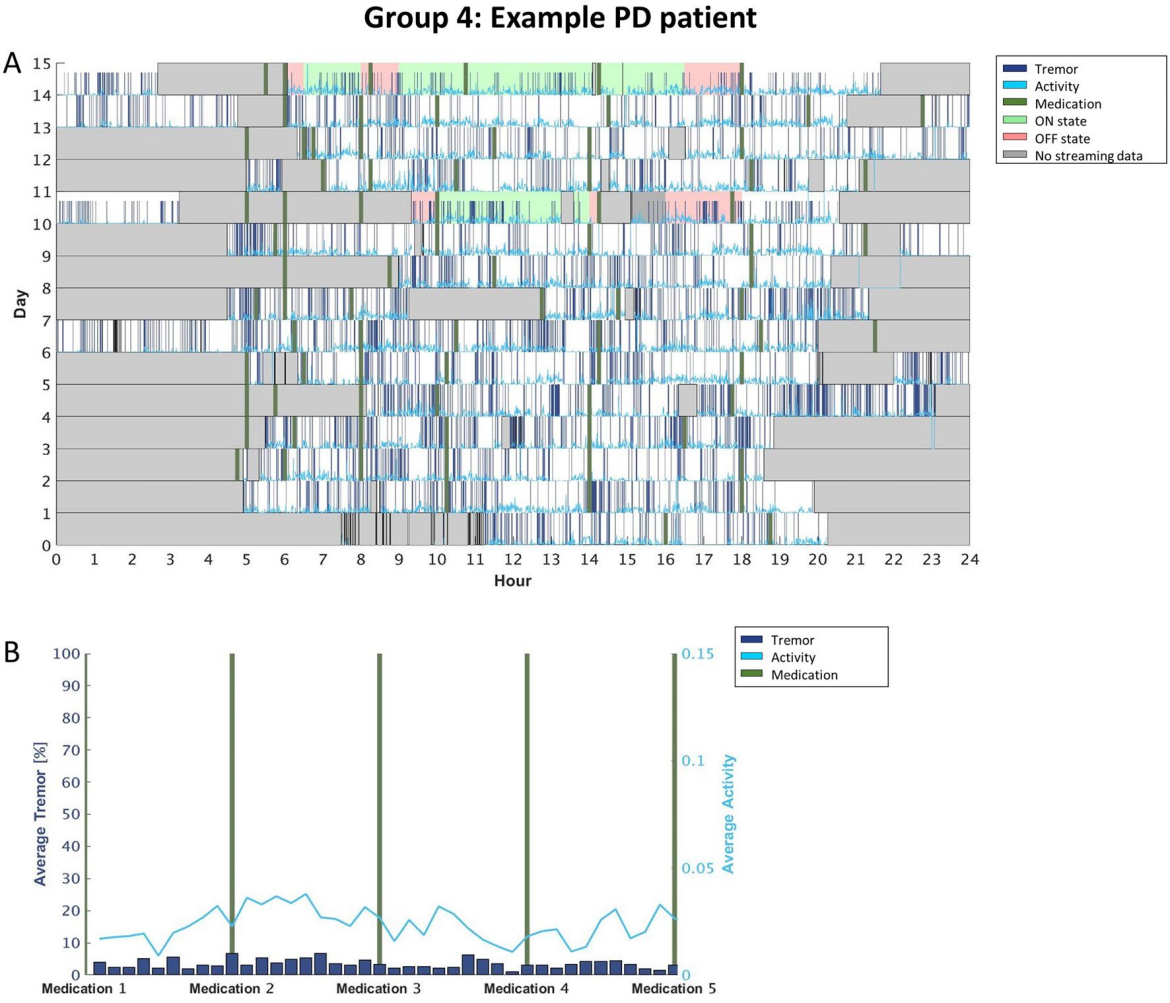
Group 4: A participant not showing any regularity in tremor fluctuations throughout the days of the experiment. (**A**) The raster plot, (**B**) the averaged tremor and activity level scores when scaling the medications intake times. The tremor did not fluctuate around the time of medications intake. Also the activity level appeared relatively constant and it was quite low across the day and during the whole HBM period.

While visually inspecting the data, we observed that individual tremor fluctuations could be categorized in 4 different patient groups. Group 1 includes participants that exhibited tremor fluctuating according to their medication intake (i.e., the SWA recorded tremor mainly around the time participants reported taking the medication when they were presumably OFF; n = 6; see Fig. 2). Group 2 included participants that systematically experienced tremor mainly in a specific part of the day, independently of the medication cycle (e.g., more tremor in the afternoon than in the morning; n = 2; see Fig. 3). Group 3 consists of participants with constant tremor throughout the day (n = 8; Fig. 4). Finally, Group 4 included participants that did not show any consistency in tremor-related fluctuations (i.e., their tremor-related fluctuations did not show any regularity across the days of the HBM; n = 5; Fig. 5).

Interestingly we saw variability also in the levels of activity but not necessarily related to tremor presence. In the participants for which tremor followed medication intakes, the activity levels increased when the tremor was lowest (so basically during ON periods, as expected; Fig. 2). We also observed cases in which the activity levels were higher when tremor was less present, even though the fluctuations did not follow medication intakes (Fig. 4). For the rest of the participants, there was no relation between tremor and activity levels (Figs. 3, 5).

**Figure 6:**
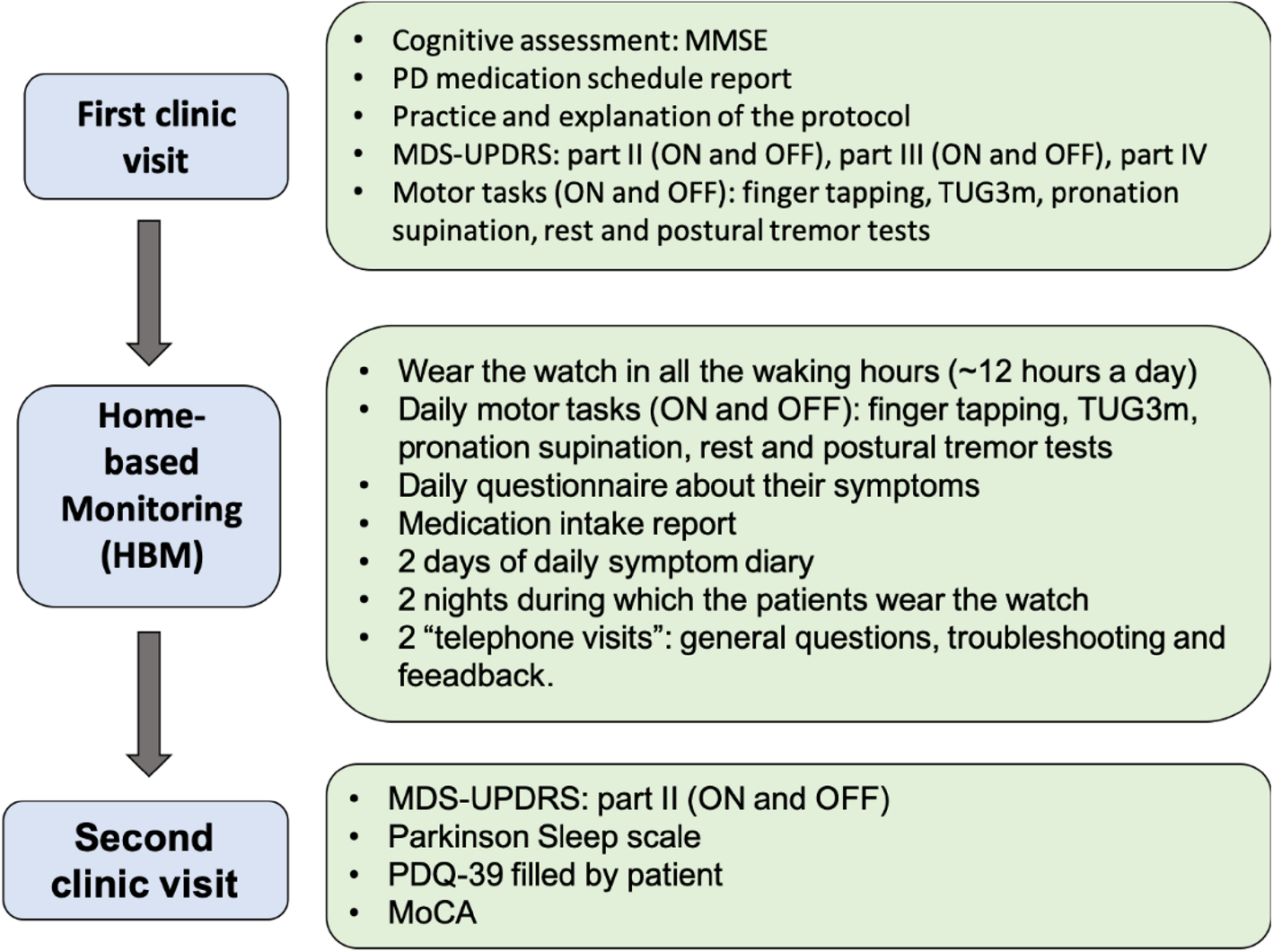
Experiment flow chart: the experiment started with a clinic visit during which participants received their kit for the HBM (i.e., AW and iPhone with the Intel App installed), were explained about the protocol, went through various cognitive and motor tests (e.g., MMSE, MDS-UPDRS) and performed a series of motor tasks; it continued with the 2 weeks of HBM in which participants were required to follow the HBM protocol described in the figure. The experiment ended with another clinic visit in which participants answered several questionnaires and returned the HBM kit.

As already mentioned, Figs. 3, 4, 5, 6 report four examples of tremor and activity level patterns of fluctuations across the 2-week HBM period representing the aforementioned 4 ‘tremor’ groups. In each figure, in the top panel, the daily tremor detection and activity level are depicted, while the bottom panel shows the averaged tremor score and average activity level score while scaling the medication intake times.

More specifically, in the top panel of each figure, the x-axis represents the time in hours (from midnight to midnight), and the y-axis represents the day of the experiment (from day 1 until the last day for each participant). The bold green vertical lines represent the time the participants took their medications every day. The dark blue lines depict all the instances during which the free-living algorithm detected tremor. The light blue line shows all the instances during which the free-living algorithm detected some level of activity. Gray areas delineate the time periods the participants did not wear the SW during a given day; therefore, the SWA did not collect any signal. The 2 days that have a colored background are the days the participants filled the daily symptom diary: Green background means the participant reported (s)he was in ON state at that time, and red background means the participant reported (s)he was in OFF state.

In addition, for increased clarity, the bottom panel of each figure depicts the scaled medication intake times of the entire experiment, while dividing the times between 2 consecutive medications in 10 bins. Then, for each bin, we calculated the percentage of time the SWA detected tremor and the average activity level for that participant. Finally, we averaged the score of each bin (of the tremor and activity level separately) through all the days of the experiment (e.g., the first bar in the figure presents the average tremor score calculated in the first bin after the first medication for all the days of the experiment). In the figure, the x-axis represents the periods of time between medications, the left y-axis represents the average tremor score and the right y-axis represents the average activity level score. Similar to the top panel, the dark blue bars are the average tremor score, the light blue line is the average activity level score and the green lines represent the medication intake time.

## Discussion

The current study aimed at further characterizing the dynamics of motor symptoms, including tremor, dyskinesia, and activity levels, during 2 weeks of continuous home-monitoring in 21 advanced PD patients who experienced significant MFs. Data were collected using the Intel^®^ Pharma Analytics Platform, comprised of an SW and a dedicated app (SWA), and provided additional validation that accelerometer and gyroscope based data and digitally assessed active tasks from a consumer SW can capture daily fluctuations in motor symptoms.

First, we showed high compliance with the protocol in all of its aspects in line with previous studies in the PD population using the same platform^24,25^.

However, correlation analyses between the SWA scores extracted via dedicated algorithms for tremor and dyskinesia, and both the clinician assessments during Visit 1, and the MDS-UPDRS scores for these symptoms, showed significant results only for tremor.

The correlation of SWA tremor score and the clinician’s tremor score were better in the OFF time than in ON time, probably due to the fact that the SWA score was mainly based on tremor occurrence and not tremor severity; thus decreased tremor amplitude as occurs in the ON state does not show in the SWA score as it does in the clinician score. Additionally, the relatively small sample size, may contribute to the low correlations. Further, the relatively weak to moderate correlation values between the SWA objective measures and clinical scores obtained from the UPDRS parts 2 and 3, may stem also here from the fact that SWA tremor score was based on tremor occurrence and not tremor severity. Moreover, the SWA scores perhaps reflect more truly the day to day objective symptoms, than the subjective experience or the single evaluation of the patient by the clinician. False assignment of “dyskinesia” in patients that did not exhibit them could be an additional problem. To what extent the SWA measure truly reflected dyskinesia warrants further investigation.

In this study we aimed to exclude patients with levodopa-resistant tremor, based mainly on a single on-site clinical assessment in the OFF and in the ON medicated state. Yet still the SWA algorithm detected “ON” tremor. This may be because a single assessment may have not reflected the true occurrence of tremor during the day(s). Additionally, SWA tremor score was based mainly on tremor occurrence and not on tremor severity, thus potentially underlying the reported weak to moderate correlation between sensor measurement and clinical assessment. This may also explain why the SWA tremor score and the clinician's tremor score were better correlated in the OFF time than in ON time; in other words, decreased tremor amplitude as predominantly occurring in the ON state would manifest in the clinician tremor score but not in the SWA score.

Furthermore, when correlating participants’ answers provided in the 2-days diaries (i.e., about their subjective feelings of being in OFF/ON states) with the SWA data, we found that, as expected, tremor was detected significantly more in self-declared OFF state. In line with the expectations, we also observed that activity levels were significantly higher in self-declared ON states. SWA dyskinesia tracing did not show an increase in the self-declared ON vs OFF states. However, dyskinesia can be a problematic measure for ON/OFF state estimation since it can also capture intentional fidgeting like finger tapping or foot knocking. In accounting for these results, it should be mentioned that patients recruited to this study had advanced PD with discernable ON and OFF episodes. However, as seen in Fig. 1 and in Table 4, the SWA detected a significant increase in tremor in 38% of the patients when in the OFF state. This is again probably due to the fact that the tremor was scored by the algorithm based on the time it was present and not based on its severity, which surely helps to define patients' motor state. While the activity level was higher in the ON state in most patients, this was only significant among 45% of the cohort. Overall, these results suggest that the algorithms for extracting tremor and activity levels were more reliable than those for dyskinesia. Alternatively, it is also possible that patients are more reliable at reporting ON and OFF states as indicated by tremor and less aware of ON and OFF states indicated by dyskinesia. Yet, in summary, to further characterize individual MF profiles, we focused on these last two parameters with the final aim of characterizing their fluctuations as a function of medication intakes.

We observed that less than a third of our PD sample (n = 6) exhibited an l-dopa-responsive tremor, namely tremor varying according to the medication cycle (i.e., increasing in OFF, and improving in ON; see Fig. 3); these patients represent the ‘classic’ tremor fluctuations’ dynamic^16^. For the remaining participants, l-dopa treatment either did not have a consistent beneficial effect on tremor or no effect at all (Figs. 4, 5, 6). In other words, through this 2-week HBM, we were able to objectively differentiate between PD patients with tremor responding to l-dopa, and those for which tremor could not serve as a marker for medication-associated MFs. Most interestingly, in certain patients, our results could set grounds for l-dopa dose augmentation or changes in their intake’s schedule: for instance, the patient’s tremor signature depicted in Fig. 4. showed that in the mornings the tremor symptom tended to follow the medication cycle, but in the afternoon the patient tended to experience persistent tremor, independently of the medication cycle. Thus, from this outcome, clinicians could conclude that this specific patient may need to re-adjust his/her levodopa intake/dosage during the afternoon.

Similarly, also activity levels may provide interesting insights into the medication cycle dynamics and participants' habits. Figures 3 and 5, for instance, depict two participants that showed more activity between medication intakes, during the ON periods, (Fig. 3) or selectively when the tremor was not present (Fig. 5). Figures 4 and 6, however, showed activity levels that were not modulated by medication intakes nor tremor presence. Most participants show a decrease in activity levels towards the end of the day, most probably highlighting personal habits. The current data, therefore, suggests that monitoring of activity is an important aspect to capture during HBM as it can provide clinicians with more comprehensive information about the lifestyle of patients. Such data could be used to improve/direct to individualized rehabilitation strategies. Note however, that while our results suggest that the level of activity is important and can serve as an indirect reflection of the motor condition, the current data do not confirm that the Intel measure of activity is a good reflection of bradykinesia (as originally conceptualized). Indeed, bradykinesia is the main l-dopa-responsive symptom of PD, but our data did not show consistent activity and l-dopa fluctuations. This is a limitation of the Intel algorithm.

The current study represents our first attempt to characterize personalized MF dynamics in advanced PD, confirming that MF properties in this population, are not a “one shoe fits all” scenario. These initial data, capturing individual dynamics of tremor-related and activity-related fluctuations, highlight the natural variability of PD patients’ symptomatology, pointing to a need for more research on the crucial topic of HBM protocols with wearable sensors.

### Comparison with other methodologies for monitoring at home activities in PD

Several home monitoring systems are available for persons with PD. For example, the PDMonitor^®^ is a system intended to be used for continues home monitoring of motor related symptoms of PD^26^. PDMonitor demonstrated high correlation between the severity of the majority of the symptoms and the clinicians’ evaluation. Nevertheless, this system consists of 5 sensors and hence is more cumbersome as compared to the SWA. Another device is the STAT-ON that provides information about motor symptoms, gait and ON/OFF stated^27^. The relative disadvantage of STAT-ON is that it does not measure tremor which is one of the most common symptoms of PD. Additionally, the Kinesia360 continuously monitors motor symptoms via wrist and ankle sensors^28^ and demonstrated relatively high correlations with clinicians evaluations. Compared to all these sensors, SWA described in the present study is incorporated within a device which is primarily a SW with regular daily amenities such as fitness tracking general health related capabilities and, of course, wireless tele communication.

In addition to the mentioned systems, previous work used similar protocols to measure MF^16,21,22^. These previous works, however, mainly focused on validating the HBM approach and the reliability of SW sensors to extract expected MF properties correctly, e.g., recording more tremor in OFF than in ON^16^. This approach, albeit obviously needed to validate the methodology, entirely disregarded the characterization of individual differences. Such differences are nonetheless, the most crucial innovation potentially introduced by the HBM approaches with wearable sensors. Indeed, the final aim of these protocols should be to allow the capture of the unique symptoms’ signature of each PD patient, eventually enabling the tailoring of the treatments to each specific profile with high level of accuracy.

### Limitations, implications and future directions

This study provides useful insights into symptoms and fluctuations in activity and symptoms throughout the day. However, the current results are not fully conclusive and reliable. In particular, the estimation of dyskinesia did not prove to be robust. As for tremor, the correlations with clinical assessment were not sufficiently high. On the other hand, this study demonstrates the ability to identify individual ‘signatures’ of the daily cycles of the tremor occurrences with respect to medication consumption, and activity levels (compare Figs. 2, 3, 4, 5). Yet, the low number of participants prevent us, at this point, to define clear sub classifications with the PD cohort.

As discussed above, there are some limitations to the use of the SWA. Importantly, it is obvious that an inertial measurement unit (IMU) that is located only in one place on the body (i.e., wrist) is limited in providing a comprehensive picture of the kinematics of a patient, including, e.g., gait and balance functioning, or information about tremor or dyskinesia severity in different body parts. Furthermore, gait and balance related impairments, e.g., freezing or balance lost, are not addressed. Multiple sensors recording multiple motor behaviors in diverse body sites can enable a more detailed personalized sensing approach.

Our work, however, in particular the analytical approach (e.g., daily symptom fluctuations—c.f. Figs. 2, 3, 4, 5) is a first important step in this direction but more work is needed. For instance, future research should consider continuous monitoring of tremor severity and not just tremor presence, as was the case here, ultimately refining the results further, and the work should be extended to other PD-related motor symptoms, including dyskinesia and bradykinesia, to characterize more comprehensive individual profiles in particular to fully capture all aspects of the motor OFF and ON conditions.

An emerging approach is to train an IMU-based device with machine learning (ML) algorithms for extracting and identifying individual patient’s typical and personalized subtle clinically related features, that are not easily detectable even by movement disorders experts^29^. With continuous monitoring protocols, that rely on ML algorithms, we will be able to more precisely recognize each advanced PD patient’s specific features for ON and OFF conditions, and will enable optimal identification of the patient's motor state and daily MF. We believe this may be the key to utilizing the HBM approach at its fullest potential (provided that such multiple, smart sensors’ set-ups will be easy to wear and simple to use by participants in a fully independent manner at their home). Eventually these data sources along with artificial intelligence models may be further combined with controllers that respond to extracted data with operational suggestions via the treating neurologist or directly provided to patients, to improve the patient outcomes and quality of life.

MFs and LID are common clinical problems affecting the everyday lives of up to 80% of patients with PD^30^ contributing to progressive disability and decrease in quality of life. Continuous remote monitoring of fluctuating PD patients with wearable sensors is an advancing technology that has already been shown to provide authentic and useful information on daily OFF and ON conditions^16,19–24^. In a few of these studies improved decision-making processes and patient outcome have also been achieved^16^. Furthermore, the use of these technologies has the potential to reduce the high healthcare costs and have a facilitatory impact on the medical resources’ shortage by combining it with telemedicine and other Digital Health tools^29^. Along these lines this technological development has the potential to promote a revolution in PD clinical practices and research.

## Methods

### Participants

Inclusion criteria consisted of male or female patients, over 30 years old, diagnosed with PD, according to the Movement Disorders Society (MDS) PD diagnostic criteria^31^, for at least 5 years. Patients had to be treated with three or more daily oral l-dopa doses and report significant MFs and (preferably) also LID, and on Hoehn and Yahr stage^32^ 1–3 while ON. Patients were to report moderate to severe functional impact of fluctuations according to question 4.4 of the MDS-UPDRS^31^ score of 3 or above. Additionally, all participants were to have the ability to operate smartphone technology.

Exclusion criteria consisted of a score of less than 20 on the MMSE^33^ or report of significant cognitive decline, psychiatric impairment, or additional major comorbidity that would preclude study participation, as determined by the principal investigator. Furthermore, patients with l-dopa resistant tremor (tremor during ON), l-dopa resistant freezing (freezing during ON) or previous functional neurosurgical procedure and/or chronic treatment with levodopa-carbidopa intestinal gel infusion administered via percutaneous endoscopic gastrojejunostomy (PEG-J) tube, were excluded.

The experimental protocol was approved by the institutional review board (IRB) of the Sheba Medical Center. All methods were carried out in accordance with relevant guidelines and regulations.

### The home-based monitoring (HBM) apparatus

The home-based monitoring (HBM) apparatus used in this study consists of a SW (Apple watch, AW, series 4, 40 mm with operating system watch OS 6–7) and a smartphone (SP, Apple iPhone 8, with operating system iOS 11–14). An application, which is part of the Intel^®^ Pharma Analytics Platform, was installed in advance on the iPhone.

#### Apple watch

The AW has various sensors, such as an accelerometer and a gyroscope, the data from these sensors were sampled at a frequency of 50 Hz. The AW contained Apple’s Health Kit that collects various gait and physiological measures (step count, walking distance, heart rate, etc.) and a heart rate monitor sensor. The AW also collected passive data from the sensors analyzed by the Intel^®^ Pharma Analytics Platform using in-built algorithms (see sections below).

#### Intel^®^ pharma analytics platform

The Intel^®^ Pharma Analytics Platform is an edge-to-cloud artificial intelligence platform for continuous data collection and analysis using sensors, wearables, and mobile technologies.

Intel’s platform is a full end-to-end service that includes devices for sensory data collection, a dedicated mobile application and secured cloud storage. This includes a library of machine-learning algorithms developed specifically for PD that have the capability to detect and quantify the presence of various PD-related symptoms. The platform is device-agnostic and supports multiple types of medical and consumer grade devices. Beyond wearables, the platform supports data collection from sensors that are placed at the patient home.

All sensor data are collected in a stringent, secure, fault-tolerant manner. All data are securely stored in the cloud and are de-identified. The Intel platform does not collect any Potentially Identifiable Information (PII). Intel applies highly strict security, privacy and quality management procedures and complies with the healthcare industry standards as well as regulations (e.g., General Data Protection Regulation).

#### Algorithms

The Intel platform is equipped with a compendium of algorithms to extract clinical insights from the raw sensor data. These solutions enable the tracking of disease-specific symptoms as well as Quality of Life (QoL) parameters. The algorithms were validated to quantify PD tremor, bradykinesia, and dyskinesia in various tasks and everyday life situations^34–36^. More specifically, two different algorithms were used in this protocol for HBM of PD symptoms: (1) the free-living algorithm and (2) the motor tasks algorithm.

The free-living algorithm evaluates the tremor, dyskinesia, and activity level separately and continuously throughout the day (i.e., whenever the participant wore the watch). The tremor and dyskinesia free-living algorithm samples the raw accelerometer data every 30 s and assigns a score (either 0 or 1) for each symptom. A score of 0 indicates an absence of the symptom (tremor or dyskinesia) in the past 30 s, while a score of 1 indicates that the algorithm detected the symptom within the past 30 s. The activity level free-living algorithm samples the raw data every 5 s and assigns a score between 0 and 1 that evaluates the participant's level of activity in the past 5 s.

The motor tasks algorithm evaluates the tremor, dyskinesia, and bradykinesia while the participants perform their daily motor tasks (see “Experimental protocol”). At the end of the motor task, the algorithm assigned one score for each symptom, which refers to the severity of that symptom during the whole motor task. The motor task scores, termed smartwatch and algorithm (SWA) scores, ranged between zero and two (see the equivalent clinician score in Table 7).

**Table 7:** SWA score vs clinician's score.

SWA score	Clinician score
0	0
1	1
2	2 and above

The SWA range of scores (left column) provided by the motor tasks algorithm together with the clinician’s range of scores (right column) supplied during the in-clinic motor tasks (see *Experimental protocol* section for further details). The clinician scores ranged between 0 and 4, but we slightly modified them for analysis purposes to make them comparable to scores provided by the SWA.

#### Smart phone and mobile application

Intel's platform includes a customizable mobile application. The mobile app was designed by usability experts and was previously tested with patients to ensure ease of use by an elderly population with PD and already used in multiple international studies and clinical trials^24,25,34,37^. The mobile app supported translations to various languages and was presented in Hebrew in this study.

The application includes several modules, which allow the collection of passive sensor data using the free living and motor tasks algorithms described above, electronic patient-reported outcomes (ePROs). ePROs are captured with electronic daily symptom diaries, daily questionnaires, and reports on the ongoing medication stage, guided structured motor tasks, and medication intake reminders and reporting (based on the participant's medication schedule)—see section *Experimental protocol* below for details on all the measures collected during the experimental procedure.

### Experimental protocol

Each patient attended two clinic visits, the first of which was before and the last after the 2-week HBM period using the SWA and patient-related outcomes (see Fig. 6 for a summary of the experimental protocol).

#### First clinic visit

During this visit, the neurologist provided the patients with a comprehensive review of the study purpose and methods and the anticipated patient obligations, followed by their signing of the informed consent form. Medical and PD-related history, as well as the exact present PD medication schedule, are recorded. For inclusion, patients underwent a cognitive function screening using the Mini-Mental State Examination (MMSE)^33^ for inclusion. Then the participants were observed and assessed, in OFF and ON states, using the MDS‐UPDRS^2^, part II (motor activities of daily living) and part III (motor examination), during the ON-state and the OFF-state, and part IV (motor complications), to confirm the presence of significant, recognizable MFs (i.e., one of the inclusion criteria for the study) and LID. Participants were also trained on the experimental protocol, data acquisition, and input and handling of the study device. This was followed by supervised in-clinic motor tasks during OFF and ON conditions (as to be done daily during the HBM period) wearing the SW. These in-clinic motor tasks had two functions: training participants on the tasks they were required to repeat daily during the HBM period (see Fig. 1) and collecting in-clinic ratings (of tremor, dyskinesia, and bradykinesia and gait) by the neurologist.

#### The motor tasks (both in-clinic and during the HBM period)

1. S**tatic postural test**—participants must outstretch their hands for 30 s (SWA measures tremor and dyskinesia).
2. **Static rest test**—the participants are required to sit with their hands resting on the armrest for 30 s (SWA measures tremor and dyskinesia)
3. **Pronation-supination**—the participants must stretch the hand with the watch forward and rotate their wrist as fast as possible for 15 s (SWA measures bradykinesia).
4. **Finger tapping**—performed on the iPhone Intel system app. In this task, the participants must tap on the circles that appear on the app as fast as possible, once with their right hand and once with their left hand (SWA measures the time between each tap and the number of valid taps (i.e., taps inside the circle).
5. **Timed up and go** (TUG)—the participants are required to rise from a chair, stand up, walk 3 m, turn around, walk and sit back on the chair (SWA measures the time it takes the participant to complete the test).

The neurologist rated the participant's tremor, dyskinesia, and bradykinesia using a standardized scale (see Table 1).

The participant's PD medication schedule was fed into the mobile application so the participants would receive reminders to take their medication according to their exact schedule. The participants were to report when taking the medication on the app.

At the end of the clinic visit, the participants take the devices (iPhone and SWA) home and begin the 2-week HBM period, and a time for visit number 2, 2 weeks later, is set.

#### Home-based monitoring (HBM) period

During this 2 weeks phase, participants were required to wear the SWA 12-h per day and to communicate daily with the Intel HBM App (see Fig. 1).

Each day, patients were required to perform motor tasks (described in the previous section), both in the ON state and OFF state, and to answer short questions about their symptoms. These questions popped up on the mobile phone app at different times every day, asking the participants to rate their symptoms (tremor and dyskinesia) and state (ON or OFF) in the past 10 min. Patients had to report each medication intake on time. In addition, they were required to fill a 12-h (from 8 AM to 8 PM) ‘PD daily symptom diary’ on two non-consecutive days, assigning the following motor states—ON, ON with dyskinesia, OFF, or asleep, every 30 min.

Two conditions had to be met to end the HBM period. The first was to complete 14 daily motor tasks in the ON state and OFF state (totaling 28 motor tasks). The second condition was to have complied with the request of two self-written, complete daily symptom diaries (we considered a ‘complete diary’ if the participant filled at least 75% of the required time windows within a maximum of 2 h delay in the reporting). Participants who did not meet these requirements within 14 days extended their experiment until completing the required tasks.

#### Second clinic visit

At the end of the HBM period, the participants visited the clinic to return the SWA and mobile phone and for an additional short assessment. The neurologist evaluated participants using the MDS-UPDRS questionnaire (part II regarding ON-state and OFF-state and part IV). Patients were assessed additionally regarding cognitive functions and filled-in questionnaires regarding sleep quality, daily sleepiness, and quality of life, using the Montreal Cognitive Assessment, MoCA^38^, the Parkinson's disease sleep scale (PDSS2)^39^, the Epworth Sleepiness Scale (ESS)^40^ and the Parkinson’s Disease Questionnaire (PDQ-39)^41^, respectively.

### Data analysis

#### Compliance with the study protocol and medication intake

We measured the adherence to protocol of the participants in four ways: (1) overall use of the SWA, (2) delay in symptoms report collected from the daily questionnaires and the daily symptom diaries, (3) motor tasks completion, and (4) medication intake report and medication intake compliance. For a detailed description of the computed analyses, see Supplementary Materials.

#### Algorithm validation (SWA vs clinician)

To validate the SWA outcomes, we considered data collected during the first clinic visit (i.e., before the beginning of the HBM). Specifically, we correlated the scores assigned by the SWA during the practice of the daily motor tasks performed during the visit and the scores to these same motor tasks assigned by the clinician (for more details, see Supplementary Materials).

#### MDS-UPDRS and sensor-data correlations

During the first (i.e., before the start of the HBM period) and second clinic visit (i.e., at the end of the HBM period), the clinicians evaluated participants using the MDS-UPDRS questionnaire^2^. Spearman correlations were calculated between the overall tremor, dyskinesia and activity scores during free-living conditions measured by the SWA, as well as tremor scores also measured by the SWA obtained during the motor tasks (see section *Experimental protocol*) with the MDS-UPDRS scores rated by clinicians (Items # 2.10, 3.15, 3.16, 3.17, 4.1, 4.3, 4.4 vs the SWA tremor scores, items # 4.1, 4.2, 4.3 vs the SWA dyskinesia score, items # 4.1, 4.2, 4.3, 4.4 vs the SWA activity score).

Specifically, the amount of daily tremor was derived from the tremor of the free-living SWA, which detected either the absence (scored as 0) or presence of tremor (scored as 1) on the participant’s wrist every 30 s. The daily tremor prevalence was then calculated as the percent of time during which tremor was detected by the SW each day. The patient’s overall tremor was computed as the average of all their daily tremor prevalence scores.

Similar calculations were used to assess the overall dyskinesia of each participant. Similarly, the overall activity for each patient was calculated as the average of all daily activity scores (as described in the section *Algorithms*).

Finally, the overall motor tasks tremor score derived from the SWA motor tasks' algorithm was calculated as the average of all tremor scores collected during all daily motor tasks that each participant performed, separately for OFF and ON states (see the sections on the clinical and daily motor tasks for further details).

#### Daily symptom diary

The participants filled out a daily symptom diary for 2 days during the HBM period. The participants were required to report their state (ON, ON with dyskinesia, OFF, asleep) every 30 min, referring to the past 30 min. We calculated the tremor, dyskinesia and activity level for every report entry as recorded by the SWA. Specifically, the tremor and dyskinesia were calculated as the percent of time the SWA recorded each of the symptoms (during the 30 min slot), and the activity level was calculated as the average activity level score measured by the SWA during the 30 min slot.

Unpaired *t*-tests were performed separately for each SWA measure (tremor, dyskinesia and activity level), comparing the presence of the aforementioned symptoms in slots the participants reported they were in ON state vs slots the participants reported they were in OFF state.

#### Daily home motor tasks in OFF and in ON

Every day of the HBM period, participants performed a series of motor tasks (see section *The motor tasks (both in-clinic and during the HBM period)* for a description of each task). Each task was performed twice, once during the ON and once during the OFF state. At the end of each task, the participants reported in the smartphone app whether they were ON or OFF their l-dopa medications during the performance.

For each daily motor task, an overall OFF and ON score was calculated as the average of the scores collected during all the tasks performed by each patient.

T-tests were performed between the SWA score of each test in ON vs OFF conditions (according to the participant's report) to quantify the benefits of being ON medications in these tasks. The t-tests were conducted on the following variables: tremor—average tremor score for each participant (derived from the sum of the rest and postural tremor scores that the motor tasks algorithm provided for each task); finger tapping—average time between taps in the finger tapping test (calculated as the average time of all taps for each motor task separately); the number of valid taps (taps inside the circle) for the finger tapping test; TUG 3m test—average time of the TUG3m test.

Finally, we also wanted to obtain a measure of the variability of the scores for each participant in each motor task during the whole HBM period. To this end, we calculated the average score and the standard deviation (SD) for each task (i.e., for each relevant dependent variable collected in each task, as described in the paragraph above) and for each participant separately. We considered as a signature of variability all scores that differed ± 1 SD from the average score for that individual in that given task/per dependent variable.

#### Individualized motor fluctuations’ patterns: raster plots

One inclusion criterion for participation in the study was the presence of MF. Even though PD-related motor symptoms are traditionally expected to fluctuate with the medication cycle, in practice these symptoms often fluctuate differently among patients. Therefore, to be informed about the individual trend of fluctuations throughout the day, one needs to consider each participant separately. To obtain an individualized signature of the MF of our participants, we extracted from our data individual variations in tremor and activity levels.

Specifically, data are presented by creating individual raster plots that depict the collected SWA scores for the two aforementioned measures throughout the days of the experiment by Intel's free-living algorithms. We added to the raster plots the times the participants took their medications (according to their report) and their daily symptom diary reports. Additionally, in order to improve the visualization of participants' symptom fluctuations according to medication schedule, we averaged data over the days of the HBM and scaled the time according to medication intakes. This can provide a clearer overview of the individual variations in the tremor experienced by each patient during the day, and the activity level can allow us to further characterize the extent to which l-dopa medications affect the patients’ daily lives.

### Supplementary Information

Supplementary Information.

## Data Availability

The data sets generated and analyzed in this study are available from the corresponding author upon reasonable request.
