# Supplementary material for: Home-based monitoring of persons with advanced Parkinson’s disease using smartwatch-smartphone technology
Source: Sci Rep. 2024 Jan 2;14:9. doi: 10.1038/s41598-023-48209-y (PMC10761812; doi:10.1038/s41598-023-48209-y)
Supplement: Supplementary file 1 — Supplementary Information. [file 41598_2023_48209_MOESM1_ESM.docx]

**Home-based monitoring of persons with advanced Parkinson's disease using smartwatch-smartphone technology**

**Supplementary Materials**

Tsviya Fay Karmon^1^, Noam Galor^2^, Benedetta Heimler^2^, Asaf Zilka^1^, Ronny P. Bartsch^3^, Meir Plotnik^2,4,5^, Sharon Hassin-Baer^1,6^

^1^Department of Neurology, Movement Disorders Institute, Sheba Medical Center, Ramat Gan, Israel

^2^Center of Advanced Technologies in Rehabilitation, Sheba Medical Center, Ramat Gan, Israel

^3^Department of Physics, Bar-Ilan University, Ramat Gan, Israel

^4^Department of Physiology and Pharmacology, Sackler Faculty of Medicine, Tel Aviv University, Tel Aviv, Israel

^5^Sagol School of Neuroscience, Tel Aviv University, Tel Aviv, Israel

^6^Department of Neurology and Neurosurgery, Sackler Faculty of Medicine, Tel Aviv University, Tel Aviv, Israel

**Methods**

**Data analysis**

Compliance with the study protocol and medication intake

(1) Overall use of the watch was measured as the average number of hours the participant wore the watch per day (participants were instructed to wear the watch 12 hours per day). We also calculated whether the hours the participants wore the watch decreased as the HBM study period progressed.

(2) Symptoms were reported by participants in the daily symptom diaries that participants filled out for 2 days during the HBM period and in the daily questionnaires. To evaluate the compliance with the daily symptom diary reports, we checked whether the participants answered the questions on time (i.e., every 30 minutes) and calculated the average delay time (we consider a delay in reporting as more than 30 minutes after the planned time, i.e., basically whether they skipped one report). To evaluate compliance with the daily questionnaires, we calculated the number of days participants skipped the questionnaire.

(3) Motor task completion- the participants were required to complete the motor tasks every day (the condition to end the experiment was to have at least 28 motor tasks, 14 in OFF state and 14 in ON state). The compliance, in terms of the motor tasks, was measured as the number of skipped motor tasks (until the participant reached the number of 14 complete motor tasks).

(4) Medication report compliance was monitored by collecting two types of information from participants. First, we checked whether the participants reported that they took the medication or not. Second, we checked the delay in reporting medication intake, i.e., the time delay between when they recorded in the app that they took the medication and the time they actually took it, according to their own report. We considered it to be a delay if the time of reporting was 30 minutes or more after it was taken (a skipped medication wasn't considered for the delay analysis).

Compliance with the medical treatment- The medication intake was measured as the percent of medications the participant did not take at all, and as the percent of medications, the participant did not take at the correct time (the correct time was considered as 30 minutes before or after the planned time).

Algorithm validation (AW vs clinician)

In the first clinic visit (i.e., before starting the home-based monitoring period), the participants performed in-clinic motor tasks during ON and OFF states (see experiment protocol). We used these in-clinic motor tasks to perform Spearman correlations between the AW measurements and the clinician's scores (see Table 1 in the main document for comparing the scores).

Rest tremor and postural tremor- Spearman correlations were calculated for each motor task between the AW tremor score and the clinician score (see Table 1 in the main document).

Tapping test- 2 correlations were calculated: between the clinician bradykinesia score (assigned during the clinic motor task) and the number of taps recorded, and between the same clinician bradykinesia score and the average time between taps.

**Results**

Compliance with the study protocol and medication intake

(1) Overall use of the watch: The average time the participants wore the watch per day was 13.6 hours (SD= ±2.32). 81% of participants wore the watch > 12 hours per day (mean 14.52, SD= ±1.19) while the remaining 19% of participants wore the watch < 12 hours per day (mean 9.65, SD= ±1.68) (see Table S2). Over the HBM period, we did not observe any significant decrease in the hours the participants wore the watch (fluctuation range: 11-17 hours, mean 13.6 ± 2.3 hours) (see Fig. S1).


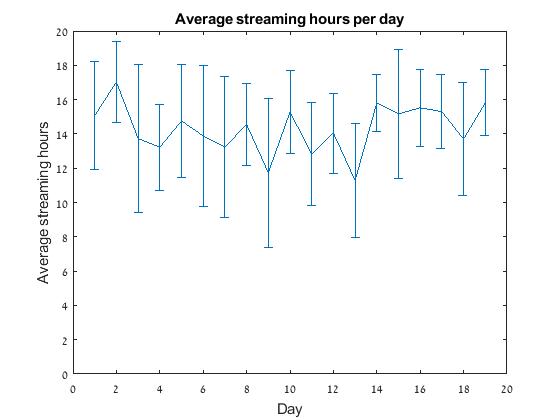


**Figure S1.** The average hours per day the participants wore the watch across the HBM period.

(2) Patient reports:

*a. Daily symptom diary reports*: 71% of participants had, on average, less than 30 minutes delay in filling the diary (mean= 12.67 minutes, SD= ±6.7; high compliance). The remaining 29% of participants had an average delay longer than 30 minutes (mean= 89.67 minutes, SD= ±55.15; low compliance) (see Table S2)

b. *Daily questionnaires*: 76% of participants skipped either 1 or 0 daily questionnaires during the whole HBM phase of the experiment (i.e., high compliance), and 19% skipped between 2 to 4 daily questionnaires (i.e., medium compliance). 19% of participants skipped between 2 to 4 daily questionnaires (i.e., medium compliance), while 1 participant skipped 17 daily questionnaires (the HBM period for this participant lasted 27 days rather than 14 days as originally scheduled; low compliance) (see Table S2).

(3) Daily motor tasks completion: 43% of participants completed all the motor tasks on time, within the 2-week period (i.e., high compliance), another 43% of participants did not complete 1-2 motor tasks on time (i.e., medium compliance), and the remaining 14% of participants did not complete more than 3 motor tasks on time (average skipped motor tasks 7, SD= ±3.6; i.e., low compliance) (see Table S2).

(4) Medication report:

*a. Medication intake*: 81% of participants did not fill their medication report less than 5% of all the expected times (mean= 1.1%, SD= ±1.2%; i.e., high compliance). 14% of participants did not report their medications between 5% to 10% of all the expected times (mean= 7.9%, SD= ±2.1%; i.e., medium compliance). Finally, 1 participant did not report their medication intake more than 10% (mean= 20.9%; i.e., low compliance) (see Table S2; Fig. S2).

*b. Delay in* *medication report time*: 38% of participants reported taking their medications with a delay greater than 30 minutes in less than 5% of all the reports (mean= 1.55%, SD= ±1.53%; i.e., high compliance). 29% of participants reported their medication intake with a delay between 5% to 25% of all the reports (mean= 17.85%, SD= ±5%; i.e., medium compliance), and 33% of participants reported their medication intake with a delay, in more than 25% of all reports (mean= 41.62%, SD= ±10.1%; i.e., low compliance) (see Table S2; Fig. S2).


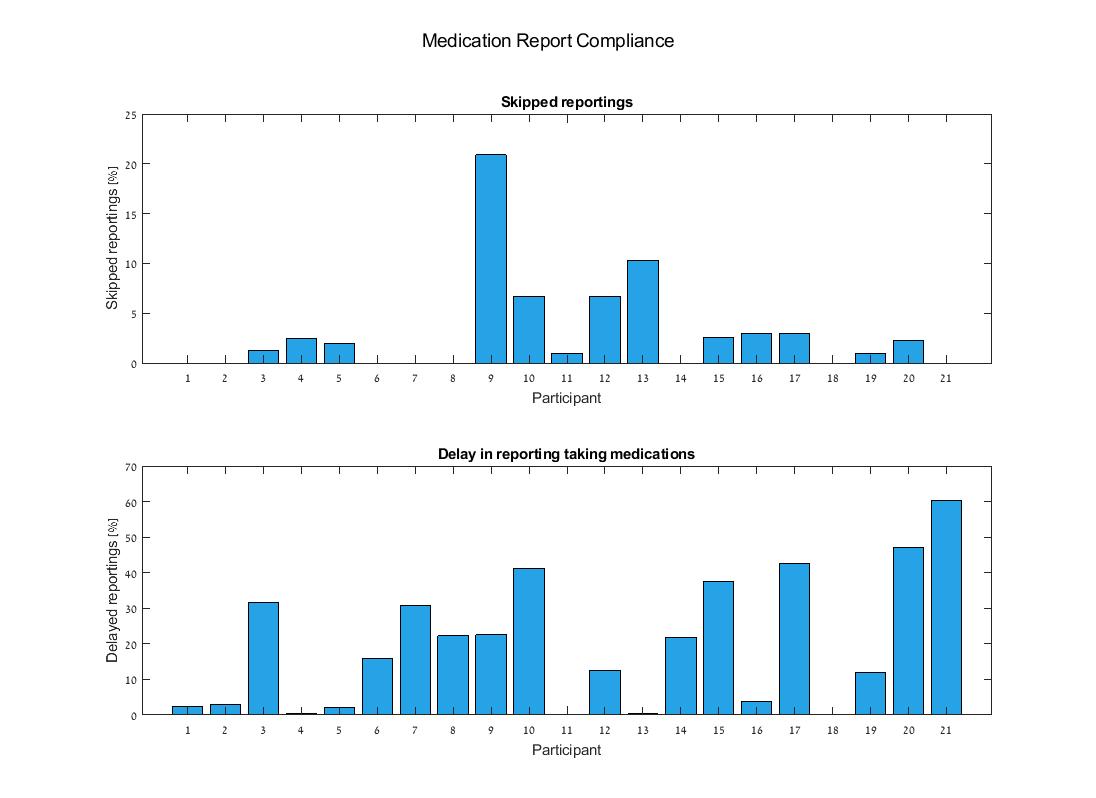


**Figure S2. Medication report compliance.** Top panel: percentage of skipped medical reports for each of the 21 participants. Bottom panel: percentage of delayed medication reports (delay > 30 minutes) in each participant.

(5) Compliance with medical treatment.

*a. Skipped dosages*: 86% of participants skipped less than 5% of all medications (mean= 1.19%, SD= ±1.06%; i.e., high compliance). 14% of participants skipped more than 5% of all medications (mean= 8.73%, SD= ±2.9%; i.e., low compliance) (see Table S2).

*b. Time of medication intake*: 33% of participants took less than 15% of all medications with a delay (i.e., >30 minutes) (mean= 6.32%, SD= ±5.31%; i.e., high compliance). 43% of participants took 15% to 30% of all medications with a delay (mean= 19.59%, SD= ±3.55%; i.e., medium compliance). 24% of participants took more than 30% of all medications with a delay (mean= 51.75%, SD= ±18.1%; i.e., low compliance).

Table S1: Summary of compliance results

| **Task** | **High compliance** | | **Medium compliance** | | **Low compliance** | |
| --- | --- | --- | --- | --- | --- | --- |
|  | Percentage of participants (N) | Mean ± SD | Percentage of participants (N) | Mean ± SD | Percentage of participants (N) | Mean ± SD |
| Overall use of watch (cut-off: 12 hours) | 81% (17) | 14.52 ± 1.19 hours | - | - | 19% (4) | 9.65 ± 1.68 hours |
| Diary report -delay (cut-off 30 minutes) | 71% (15) | 12.67 ± 6.7 minutes delay | - | - | 29% (6) | 89.67 ± 55.15 minutes delay |
| Number of skipped daily questionnaire | 76% (16) | 0-1 skipped report | 19% (4) | 2-4 skipped reports | 5% (1) | 17 skipped reports |
| Number of skipped motor tasks | 43% (9) | 0 skipped motor tasks | 43% (9) | 1-2 skipped motor tasks | 14% (3) | 7 ± 3.6 skipped motor tasks |
| Number of skipped medication report | 81% (17) | 1.1 ± 1.2% skipped reports | 14% (3) | 7.9 ± 2.08% skipped reports | 5% (1) | 20.9% skipped reports |
| Number of delayed medication reports (delay> 30 minutes) | 38% (8) | 1.55 ± 1.53% delayed reports | 29% (6) | 17.85 ± 5% delayed reports | 33% (7) | 41.62 ± 10.1% delayed reports |
| Number of skipped medications intake | 86% (18) | 1.19 ± 1.06% skipped medications | - | - | 14% (3) | 8.73 ± 2.9 skipped medications |
| Delay in medication intake (cut-off 30 minutes) | 33% (7) | 6.32 ± 5.31 % medication intake delay | 43% (9) | 19.59 ± 3.5% medication intake report | 24% (5) | 51.75 ± 18.07% medication intake report |

Table S1. Compliance results reported separately for each task (for more details see text in the dedicated paragraphs).

Algorithm validation (AW vs. clinician)

**Tremor.** The correlation between the rest tremor motor task in OFF as performed during the first clinic visit and scored by the AW and the same motor task scored by the clinician during the first clinic visit, was rho= 0.74 (p<0.001). The same correlation when participants were ON their medications was rho= 0.58 (p=0.01). The correlation between the postural tremor motor task in OFF as performed during the first clinic visit and scored by the AW and the same motor task scored by the clinician during the first clinic visit, was rho= 0.64 (p<0.001). The same correlation when participants were ON their medications was rho= 0.57 (p=0.01).

**Bradykinesia.** The correlation between the clinician's bradykinesia score assigned during the clinical motor tasks as part of the first clinic visit while participants were OFF and the number of taps in the finger tapping test performed on the mobile app during the first clinic visit, also during OFF, was rho= 0.46 (p=0.04). The same correlation while the participants were in ON was not significant (rho=-0.003; p=0.99). The correlation between the clinician's bradykinesia score and the average time between taps performed on the mobile app during the first clinic visit, while the participants were in OFF was rho= 0.49 (p=0.02). The same correlation while the participants were in ON was not significant (rho=0.01; p=0.95).

MDS-UPDRS and sensor-data correlations:

*MDS-UPDRS parts 2 and 3 correlations with Apple Watch scores*

Below is a detailed description of all correlations’ results.

The correlation between the overall tremor recorded by the AW during the HBM period and the tremor score subjectively reported by participants regarding the same HBM period (item 2.10 of the MDS-UPDRS performed in the second clinic visit) during the OFF medication state was rho= 0.64 (p=0.003; Table 3 in the main document). During the ON medication state, the correlation was rho= 0.38 (p=0.104).

The correlation between the overall tremor recorded by the AW and the combined tremor score assigned by the clinicians during the first clinic visit for questions 3.15 (postural tremor), 3.16 (kinetic tremor) and 3.17 (rest tremor) of the MDS-UPDRS during the OFF-medication state was rho= 0.62 (p=0.004; Table 3 in the main document) and during the ON medication states was rho= 0.45 (p=0.05).

The correlation between the MDS-UPDRS performed in the first clinic visit, for question 3.15 (postural tremor) and the average tremor score recorded by the AW in the daily home motor tasks during the HBM period collected in the OFF-medication state was rho= 0.49 (p=0.02; Table 3 in the main document), while for those collected during the ON medication state, the correlation was rho= 0.11 (p=0.63).

The correlation between the MDS-UPDRS performed in the first clinic visit, for question 3.17 (rest tremor) and the average tremor score recorded by the AW in the daily motor tasks during the OFF medication state was rho= 0.43 (p=0.05; Table 3 in the main document), while during the ON medication state, the correlation was rho= 0.17 (p=0.47).

*MDS-UPDRS part 4 correlations with AW scores*

Below is a detailed description of all correlations’ results.

The correlations between the overall dyskinesia score and questions 4.1 (time spent with dyskinesia), 4.2 (functional Impact of dyskinesia), and 4.3 (time spent in OFF state) are rho= 0.4 (p=0.09), rho= 0.47 (p=0.04), rho= -0.35 (p=0.14), respectively.

The correlations between the overall tremor score and questions 4.1 (time spent with dyskinesia), 4.3 (time spent in OFF state), and 4.4 (functional impact of fluctuations) are rho= -0.4 (p=0.08), rho= 0.11 (p=0.64), rho= -0.07 (p=0.77), respectively.

The correlations between the overall activity score and questions 4.2 (functional Impact of dyskinesia), 4.3 (time spent in OFF state), and 4.4 (functional impact of fluctuations) are rho= 0.3 (p=0.22), rho= -0.47 (p=0.04), rho= -0.34 (p=0.16), respectively.

Daily symptom diary

**Tremor.** Tremor was recorded in all participants during the 2-days daily symptom diaries. Overall, the AW detected, on average, significantly more tremor when participants reported they were in OFF than when they reported to be in ON (p= 0.02). More specifically, we observed that in 65% of the participants, the AW recorded more tremor when they reported they were in OFF (mean= 21% tremor-detected-time, SD= ±17%) than when they reported they were in ON (mean= 12% tremor-detected-time, SD= ±1%). For 38% of these participants, this difference was significant (p<0.02) (see Fig. 3 and Table 4 in the main document).

**Dyskinesia.** Dyskinesia was recorded in all participants during the 2-days daily symptom diaries. Overall, the AW did not detect, on average, significantly more dyskinesia when participants were in ON compared to when they were in OFF (p= 0.1). Nonetheless, 65% of them had more dyskinesia at times they reported they were in ON (or ON with dyskinesia) (mean= 43% dyskinesia-detected-time, SD = ±13%) than when they reported they were in OFF (mean 31% dyskinesia-detected-time, SD= ±13%). For 61% of these participants this difference was significant (p<0.05) (see Fig. 3 and Table 4 in the main document).

**Activity.** Overall, the AW detected, on average, significantly more activity when participants reported they were in ON than when they reported to be in OFF (p<0.01). Specifically, we found that this was true for 90% of the participants (in ON mean= 0.12 AW summed scores, SD= ±0.04; in OFF: mean= 0.08, SD= ±0.03). In 45% of these participants this difference was significant (p<0.03) (see Fig. 3 and Table 4 in the main document).

Daily home motor tasks in OFF and ON:

*Rest tremor test*- The AW detected tremor during the rest tremor motor task in 66.7% of participants. For 21% of these participants, the average motor tasks' tremor score for the OFF rest tremor motor tasks (mean AW score= 1.7, SD= ±0.28) was significantly higher than the average motor tasks' tremor score for the ON motor tasks (mean AW score= 0.35, SD= ±0.4; p<0.01). For the other participants, average tremor scores did not differ between OFF and ON conditions (OFF: mean AW score= 0.35, SD= ± 0.53; ON: mean= 0.4, SD= ±0.51).

*Postural tremor test*- The AW detected tremor during the postural tremor motor tasks in 28% of participants. For 16% of these participants, the average motor tasks' tremor score when they were in OFF (mean AW score= 0.94, SD= ±0.48) was significantly higher than the average motor tasks' tremor score in the ON condition (mean AW score= 0.2, SD= ±0.28; p<0.01). For the other participants, average tremor scores did not differ between OFF and ON conditions (OFF: mean AW score = 0.44, SD= ± 0.66; ON: mean AW score = 0.28, SD= ±0.56).

*Finger tapping test*- 90.5% of participants had fewer valid taps during the OFF motor tasks (mean= 24.6 valid taps, SD= ±8.2) compared to the ON motor task (mean= 34.4 valid taps, SD= ±6.6). For 63% of these participants, this difference was significant (p<0.001) (see Table 6 in the main document).

In addition, 86% of participants had an overall longer interval time between taps during OFF (mean= 0.44 seconds, SD= ±0.13) compared to the average interval between taps during ON (mean= 0.29 seconds, SD= ±0.06). For 44% of these participants, this difference was significant (p<0.001) (see Table 5 in the main document).

*TUG3m test-* 95% of participants took longer to complete the TUG3m test during the OFF condition (mean= 21.68 seconds, SD= ±4.42) compared to the ON condition (mean= 17 seconds, SD= ±3.6). For 65% of these participants, this difference was significant (p<0.03; see Table 6 in the main document).

*Variation over time-* Table S2 shows the percent of variable scores we detected in each participant, separately for each motor task over the HBM period.

Table S2: Variation over time of the motor tasks scores

|  | Tremor | | | | Tapping | | | | TUG | |
| --- | --- | --- | --- | --- | --- | --- | --- | --- | --- | --- |
|  | rest tremor | | Postural Tremor | | Valid taps | | Time between taps | | Time | |
|  | ON | OFF | ON | OFF | ON | OFF | ON | OFF | ON | OFF |
| PD01 | 13% | 0% | 0% | 0% | 20% | 17% | 0% | 8% | 7% | 8% |
| PD02 | 8% | 0% | 0% | 0% | 17% | 8% | 0% | 8% | 8% | 0% |
| PD03 | 21% | 0% | 0% | 8% | 21% | 23% | 7% | 8% | 7% | 8% |
| PD04 | 18% | 70% | 6% | 20% | 18% | 20% | 6% | 10% | 6% | 10% |
| PD05 | 0% | 0% | 0% | 0% | 8% | 7% | 0% | 0% | 8% | 7% |
| PD06 | 27% | 0% | 0% | 0% | 7% | 15% | 0% | 23% | 7% | 8% |
| PD07 | 0% | 0% | 0% | 0% | 6% | 23% | 6% | 8% | 6% | 0% |
| PD10 | 0% | 0% | 0% | 0% | 15% | 17% | 8% | 6% | 8% | 6% |
| PD12 | 0% | 0% | 0% | 0% | 0% | 25% | 0% | 0% | 6% | 12% |
| PD13 | 0% | 0% | 0% | 0% | 0% | 7% | 0% | 7% | 11% | 7% |
| PD14 | 0% | 8% | 0% | 0% | 0% | 0% | 0% | 8% | 8% | 0% |
| PD15 | 22% | 10% | 0% | 10% | 6% | 5% | 0% | 0% | 6% | 5% |
| PD16 | 27% | 17% | 0% | 0% | 9% | 6% | 0% | 0% | 9% | 6% |
| PD17 | 0% | 7% | 0% | 0% | 15% | 20% | 8% | 27% | 8% | 7% |
| PD18 | 12% | 6% | 0% | 0% | 0% | 0% | 0% | 0% | 6% | 0% |
| PD19 | 37% | 0% | 50% | 6% | 25% | 19% | 19% | 0% | 6% | 6% |
| PD21 | 0% | 0% | 0% | 0% | 5% | 6% | 0% | 0% | 5% | 6% |
| PD22 | 5% | 6% | 0% | 0% | 0% | 6% | 0% | 6% | 5% | 0% |
| PD23 | 0% | 0% | 0% | 0% | 13% | 0% | 0% | 0% | 7% | 0% |
| PD24 | 16% | 22% | 0% | 6% | 21% | 22% | 5% | 11% | 5% | 6% |
| PD26 | 42% | 7% | 0% | 36% | 8% | 21% | 0% | 0% | 25% | 14% |

Table S2. Variation over time of the motor tasks score. The table shows the percentage of variable scores (i.e., differing ±1 SD from the average individual score) for each motor task the participant did over the HBM period. We considered a ‘fluctuating score’ when we detected more than 20% of variable scores (i.e., differing ±1 SD from the average individual score -corresponding to > 3 variable scores) in a given participant for a given motor task (in red).
